# Adenocarcinoma - success so far and the way ahead

**DOI:** 10.17179/excli2022-5939

**Published:** 2023-03-01

**Authors:** Rabab Fatima, Mousmee Sharma, Parteek Prasher, Gaurav Gupta, Sachin Kumar Singh, Monica Gulati, Kamal Dua

**Affiliations:** 1 Department of Chemistry, University of Petroleum & Energy Studies, Energy Acres, Dehradun 248007, India; 2 Department of Chemistry, Uttaranchal University, Dehradun 248007, India; 3 School of Pharmacy, Suresh Gyan Vihar University, Mahal Road, Jagatpura, Jaipur, India; 4 Uttaranchal Institute of Pharmaceutical Sciences, Uttaranchal University, Dehradun, 248007, India; 5 Department of Pharmacology, Saveetha Dental College, Saveetha Institute of Medical and Technical Sciences, Saveetha University, Chennai, India; 6 School of Pharmaceutical Sciences, Lovely Professional University, Phagwara, Punjab, 144411, India; 7 Faculty of Health, Australian Research Center in Complementary and Integrative Medicine, University of Technology Sydney, Ultimo, NSW, 2007, Australia; 8 Discipline of Pharmacy, Graduate School of Health, University of Technology Sydney, Ultimo NSW, 2007, Australia

## ⁯⁯

A histological subtype of carcinoma, adenocarcinoma is primarily conceived in mucus-producing glands that line the organs. To date, the standard therapy in the fight of adenocarcinoma includes a combination of surgery, chemotherapy, or radiotherapy. Furthermore, the adverse effects associated with the chemotherapy regimen remain a major concern. Therefore, an alternative therapy or a combination of therapies is required to lower the morbidity rate. The key to establishing a significant prognosis towards adenocarcinomas and its global economic burden is to identify those who are at greater risk and intervene in them timely. With the emergence of precision oncology, genomic medicine and gene expression profiling, overall survival has been increased in patients undergoing chemotherapy. Recent advances in high-throughput molecular assay technology, particularly gene expression microarray, are providing a better understanding to physicians to tailor the treatment options in various cancers.

Lung adenocarcinoma (LUAD) which is the leading cause of cancer-related mortality worldwide holds an exceptionally poor prognosis and a high proliferation rate. The targeting of abnormal molecular pathways peculiar to LUAD such as p13K/AKT/mTOR/ROS/BRAF/ MAPK and JAK/STAT signaling may ultimately alter the treatment paradigm and provide hope for lung cancer patients. The Alchemist (Adjuvant Lung Cancer Enrichment Marker Identification & Sequencing) trial screened and resected 309 LUAD patients in August 2018 with EGFR & ALK mutations in the early stage and addressed the importance of targeted therapy as a part of curative care (Yuan et al., 2019[19]). The integration of radiogenic data with targeted molecular therapies may help create “big data” datasets for predicting driver mutations and identifying distinct sub-visual imaging patterns responsible for LUAD onset.

An important but underrecognized mechanism of the spread of LUAD is aerogenous metastasis from clinical, radiologic, and pathological perspective. Aerogenous metastasis results in the incessant spread of tumor cells from the primary source through the airways to adjacent or distant lung tissues. Although established histological parameters are helpful in identification, a definitive diagnosis focussing on cancer genotyping to deduce multiclonality is desirable. With a 27 % prognosis and 5-year rate of survival, lobectomy can be an option for patients presenting lobar consolidation of both lungs. Considering the low incidence of nodal and distant metastasis at the time of presentation, lung transplant may be an alternative for the diffuse pulmonary-only-spread of LUAD. This again necessitates a multidisciplinary perspective involving clinicians, pathologists, radiologists, thoracic surgeons, and geneticists for adequate diagnosis. In fact hypothesizing inhalational therapy along with genomic profiling can be a potential strategy for the management of LUAD with aerogenous metastasis (Gaikwad et al., 2014[3]). The limitations of sample availability and high cost of genomically targeted therapy call for a reliable and cost-effective reflex testing that uses focused next-generation DNA/RNA sequencing. Targeted therapies hold important management implications in designing drug delivery vehicles keeping oncogenic vignettes such as anchorage-independent survival, cancer cell shedding and reattachment to distant alveoli, in mind. Moreover, imaging based computational strategies using artificial intelligence and deep learning may be applied in determining histological subtypes of LUAD for successful evasion of surgery. Identification of broad spectrum non-invasive biological markers developed using integrative serum metabolic fingerprints may help pave a robust foundation for the detection of pulmonary nodule in LUAD (Wang et al., 2022[18]).

The guidelines endorsed by Accreditation council for continuing medical education (ASCCP) for diagnosis and management of cervical adenocarcinoma in situ highlight novel excisional procedures for ruling out invasive adenocarcinoma before the recommendation of hysterectomy. The evasion of hysterectomy in childbearing females with co-testing of endocervical samples every 6 months to 3 years who wish to conceive, may establish good prognosis in handling the devastations caused by cervical adenocarcinoma (Teoh et al., 2020[17]).

High quality endoscopy with full mucosal visualization combined with biopsy sampling for histopathology is a crucial strategy to accurately and robustly detect and risk classify gastric atrophy and gastric intestinal metaplasia. The implications of lymphadenotectomy during esophagectomy however brought glad tidings during neoadjuvant therapy for adenocarcinomas of esophagus and esophagogastric junction, maximizing survival when an optimum range of nodes are resected (Raja et al., 2021[12]). A report from an international multicenter randomized phase 3 trial, done at USA, interpreted 70 % long-term survival rates in intermediate and high-risk prostrate adenocarcinoma patients receiving short-term adjuvant androgen deprivation therapy along with salvage prostrate bed radiotherapy (PBRT) followed by pelvic node radiotherapy (PLNRT).

The notorious resistance of cancer stem cells (CSC) towards chemotherapy plays a crucial role in development of invasion and metastasis in several adenocarcinomas. Albeit significant progress, molecular characterization of secreteome and proteome of CSC, like in case of pancreatic ductal adenocarcinoma (PDAC), still needs to be explored (Huang et al., 2021[5]). This conceptualizes the role of developing anti-cancer therapeutics targeting peptides and pathways inherent to CSC progression to reduce future insurgences. In fact, an amalgamation of targeted therapies and combinational immunotherapies as well as predicting the ideal timing of these therapies play a decisive role in adequate management of various adenocarcinomas.

The clinical impact of radiolabeled 68 Ga-FAPI PET/CT versus standard of care imaging in patients with primary and recurrent PDAC predict appropriate tumor stroma interaction crucial to aggressive PDACs, which generally surpass the standard-of-care morphological and metabolic imaging (Röhrich et al., 2021[13]). Standing alone, the organoid technology holds a bright future in PDAC management after receiving Gemcitabine/Nab-Paclitaxel or Folfirinox as first-line therapeutics. A precisive tool for faithful prediction of drug sensitivity in real time, organoid technology can mimic, visualize and robustly monitor crucial biomarkers and various stages of PDAC progression *in vitro*, even before the occurrence of disease (Frappart and Hofmann 2020[2]). Moreover, construction of a 3D co-culture model of PDAC organoid using niche factors like cancer-associated fibroblasts (CAF) to evaluate stromal cell cross talks among different PDAC organoid phenotypes, provide new insights for optimum realization of subtype-based and stromal-based targeted therapy (Shinkawa et al., 2022[15]). Circulating biomarkers and liquid biopsy appear to hold promise for enhancing the multidisciplinary therapeutic strategy for PDAC. The most researched blood liquid biopsy analyte is circulating tumor DNA (ctDNA), which can offer real-time and advanced insights into the molecular profile and specific properties of the tumor. Interestingly the implications of nanoparticle-based therapies against specific and effective targeting of cancer cells have garnered much attention in the past few years (Pietrasz et al., 2022[11]). Of note, materials like liposomes, nanospheres, micelles and dendrimers seem to enhance the performance of various anticancer cargo drugs and promote their delivery in a controlled and sustained manner.

Due to non-specific targeting of cancerous cells, most popular anticancer therapeutic modalities such as surgery, chemotherapy and radiotherapy remain ineffective or require higher doses of drug. Currently, there are no non-toxic approaches that address the tremendous mortality associated with deranged proliferation of tumor cells. Natural product-based compounds or phytoceuticals such as luteolin, resveratrol, quercetin, catechins and curcumin can be an attractive strategy when synergistically used with polymeric nanoparticles to suppress proliferation of tumors. Notably, nexrutine, a non-toxic natural herbal supplement extracted from the barks of *Phellodendron amurense*, demonstrated remarkable inhibition of moderately differentiated prostrate tumors *in vitro *and* in vivo* (Ghosh et al., 2010[4]). Given the pertinent onset of adenocarcinomas from mucus producing glands, a physiologically benevolent mucoadhesive targeted drug delivery system is intriguing. Such systems, designed in concert with naturally occurring or modified polysaccharides, increase the drug retention time and consequently favor the delivery of impending therapeutics in a targeted and controlled way (Pandey et al., 2022[10]).

The relative relapse of symptoms after chemotherapy followed by short survival can be managed by targeting genes and markers associated with high-risk breast cancer patients. Based on CLEOPATRA Trial which recommended Pertuzumab, the use of humanized monoclonal antibody along with Transtuzumab and Docetaxel should be considered to be the standard-of-care for patients with metastatic breast cancer, who previously have not received any treatment. Apparently, future challenge lies with the appropriate sequencing and combining cardioprotective anti HER 2/neu targeted therapies with effective adjuvant therapy (O'Sullivan and Swain 2013[9]). Furthermore, a post hoc analysis of randomized controlled clinical trial on high-risk breast cancer patients holds valuable promise on appropriating the timing of radiotherapy after mastectomy and adjuvant chemotherapy (Chen et al., 2022[1]). Due to late diagnosis and limited response to standard chemo and radiotherapy regimens, the global burden of adenocarcinoma associated mortality is rising. Deciphering potential prognostic biomarkers such as genome-wide DNA methylation-based nomogram validated by The Cancer Genome Atlas (TCGA), may help improve prognosis in several adenocarcinoma.

Due to low prevalence and late detection, complete surgical resection with adjuvant platinum-based chemotherapy remains the cornerstone for patients suffering from hepatoid adenocarcinoma of the lung (HAL). Since currently there is no cure for HAL, a thorough understanding of cellular differentiation or serum alpha fetoprotein levels needs to be carefully considered. As the complexity of HAL is daunting, spanning cell and tissue biology, high throughput experimental technologies and computational instruments may provisionally be valuable in crafting better therapeutic outcomes (Li et al., 2021[6]). An emerging and preventive hotspot in PDAC treatment is the stimulation of immunity through pyroptosis mediated by long coding RNA (lnRNA) that plays a crucial role in PDAC pathogenesis. Pyroptosis which is a caspase dependent inflammatory cell death process may be an illuminating example favoring the understanding of prognosis prediction and intricacies of tumor microenvironment involved in PDAC (Zhao et al., 2022[20]). A novel risk score model for PDAC prognosis prediction demonstrated pyroptosis and inflammasome related differentially expressed genes from TCGA database which stratified patients into different survival and immune functions for better molecular insights of this tumor (Zuo et al., 2022[21]). A preoperative volumetric ADC histogram analysis for evaluating lymphovascular space invasion (LVSI) status in stage I endometrioid adenocarcinoma (EAC) has shown a remarkable decline in multiple volumetric metrics in LVSI positive EACs (Ma et al., 2022[7]). 

Given the increasing incidence of adenocarcinoma of the esophagus, particularly in North America and Europe, the clinical practice guidelines guided by the European Society for Medical Oncology (ESMO) include clinical and pathologic diagnosis, staging, treatment, and close follow-up, as well as algorithms for effective treatment of primary and advanced stages (Obermannová et al., 2022[8]). A fairly valuable approach fusing multidetector CT, endoscopic and transabdominal ultrasound, PET and MRI may bestow complementary modalities for early detection of regional lymph node and depth of invasion in gastric adenocarcinoma (Singh et al., 2022[16]). The primary benefit of integrating various imaging techniques includes the ability to compare results from one modality with another for better visualization of target lesions for diagnosis. Molecular targeted therapies involving mutated tumor protein p53, VEGF (vascular endothelial growth factor), RATK (receptor activator tyrosine kinases), and cell cycle pathways, may provide benefit in alleviating the response. Additionally, refining new hallmarks such as non-mutational epigenetic reprogramming, polymorphic microbiomes evading immune destruction along with epigenetic sequencing can be a heuristic tool for distilling the vast complexity of cancer phenotypes and genotypes into an expansive logical science. Finally raising awareness about various adenocarcinomas in the scientific community shall lead to definitive answers in holistically managing the global burden of cancer.

With the arrival of next generation omic-derived technologies such as single cell and single nucleus sequencing along with spatial transcriptomic, potential receptor-ligand interactions and intricate tumor architechture can be easily revealed (Schreyer et al., 2022[14]). This may help unravel the next generation clinically responsive biomarkers and therapeutic opportunities for systematic management of the disease.

## Notes

Rabab Fatima, Parteek Prasher, and Kamal Dua contributed equally. 
